# Pitfalls in assessing microvascular endothelial barrier function: impedance-based devices *versus* the classic macromolecular tracer assay

**DOI:** 10.1038/srep23671

**Published:** 2016-03-30

**Authors:** Iris Bischoff, Michael C. Hornburger, Bettina A. Mayer, Andrea Beyerle, Joachim Wegener, Robert Fürst

**Affiliations:** 1 Institute of Pharmaceutical Biology, Biocenter, Goethe University Frankfurt/Main, Germany; 2 Pharmaceutical Biology, Department of Pharmacy, Center for Drug Research, University of Munich, Germany; 3 Institute of Analytical Chemistry, Chemo- and Biosensing, University of Regensburg, Germany

## Abstract

The most frequently used parameters to describe the barrier properties of endothelial cells (ECs) *in vitro* are (i) the macromolecular permeability, indicating the flux of a macromolecular tracer across the endothelium, and (ii) electrical impedance of ECs grown on gold-film electrodes reporting on the cell layer’s tightness for ion flow. Due to the experimental differences between these approaches, inconsistent observations have been described. Here, we present the first direct comparison of these assays applied to one single cell type (human microvascular ECs) under the same experimental conditions. The impact of different pharmacological tools (histamine, forskolin, Y-27632, blebbistatin, TRAP) on endothelial barrier function was analyzed by Transwell^®^ tracer assays and two commercial impedance devices (xCELLigence^®^, ECIS^®^). The two impedance techniques provided very similar results for all compounds, whereas macromolecular permeability readings were found to be partly inconsistent with impedance. Possible reasons for these discrepancies are discussed. We conclude that the complementary combination of both approaches is highly recommended to overcome the restrictions of each assay. Since the nature of the growth support may contribute to the observed differences, structure-function relationships should be based on cells that are consistently grown on either permeable or impermeable growth supports in all experiments.

The vascular endothelium, a cell monolayer lining the blood vessels, operates as a size-selective and semi-permeable extravasation filter for plasma proteins, solutes, and fluids, thus controlling tissue homeostasis^1^. Dysfunction of the endothelial barrier, *e.g*. by opening intercellular junctions, and the subsequent edema formation is a hallmark of inflammatory processes and associated with a plethora of severe diseases, *e.g*. atherosclerosis, rheumatoid arthritis, or asthma. Unfortunately, anti-edematous drugs that specifically interact with barrier-regulating processes in endothelial cells have not been described yet. Consequently, there is a great need for basic research and drug discovery programs involving both academic and industrial partners. Both, the in-depth elucidation of endothelial barrier regulation and the comprehensive screening of potential anti-edematous compounds must be pursued. Quantitative, sensitive, and high-throughput approaches are, thus, required to monitor barrier properties of endothelial cell monolayers *in vitro* mimicking physiological and patho-physiological conditions.

Two techniques have been considered as gold standard throughout the last decades: (i) Measurement of macromolecular permeation (*e.g*. of fluorescence-labeled dextran) across a vascular endothelial cell layer as direct indicator for transendothelial solute flux and (ii) determination of transendothelial electrical resistance (TER) as measure for ionic permeability through intercellular clefts^2^,^3^,^4^,^5^,^6^,^7^,^8^,^9^. Both approaches have their individual advantages and limitations with respect to their sensitivity, time resolution, artifacts, and practicability. For either type of assay, cells have to be grown on permeable filter substrates. This experimental setup provides access to the culture medium from the apical and basolateral side and allows combining both techniques in one single experiment. However, due to the very low resistance of vascular endothelial cells, TER measurements are very challenging and have not been used routinely, whereas macromolecular permeability is regarded as a robust and convenient read-out parameter.

In 1993, a new approach has been introduced by Giaever and Keese: endothelial cells are placed on the surface of gold-film electrodes and the electrochemical impedance of the cell-covered electrodes is measured^10^. This technique is referred to as electric cell-substrate impedance sensing or short ECIS. Impedance, also described as resistance to alternating currents (AC), is a complex physical quantity that is dependent on the AC frequency since the latter determines the current pathway across the cell layer (transcellular *vs*. paracellular)^11^. For leaky cell layers with low paracellular resistances, as exclusively studied here, the current will flow primarily along paracellular pathways for most frequencies as the transcellular resistance across the cell membranes is orders of magnitude higher than the paracellular one. Similar to TER readings, the measured impedance is an indicator for the ionic tightness of the cell layer. Numerical values are, however, not identical as the impedance contains contributions from the electrodes and the overlaying medium. Using impedance measurements to monitor cell-based assays brought several technical advantages like, for instance, the option to be compatible with *in situ* microscopy, to run multispot readings providing higher throughput, to be more sensitive for leaky cell layers, or to obtain supplementary information about plasma membrane morphology through capacitance readings^12^.

Soon after introduction, impedance measurements were applied for the assessment of endothelial barrier function^13^. However, a validation of impedance measurements as alternative technique to the established use of macromolecular tracers is still missing to date. Noteworthy, in the ECIS device, cells are grown on a non-permeable substrate with access to the culture medium only from the upper side, whereas classical TER and macromolecular permeability assays make use of permeable substrates. Thus, the question arises whether the different nature of the growth substrate (permeable *vs*. non-permeable) affects the outcome of the experiment^14^. A direct comparison of both approaches for one cell type under otherwise identical experimental conditions is needed for a qualified judgment. Therefore, we set up a comparative study with a widely used human microvascular endothelial cell line (HMEC). Our major objective was to pinpoint the advantages and disadvantages of the two different approaches by treating the cells with known barrier-modulating compounds: the adenylyl cyclase activator forskolin, Y-27632, which is an inhibitor of the Rho downstream effector Rho-associated kinase (ROCK), and blebbistatin, an inhibitor of myosin II. Thrombin receptor-activating peptide (TRAP) was used to increase endothelial permeability in order to test the barrier-protecting properties of these three compounds. Moreover, also the endogenous autacoid histamine, a strong inducer of hyperpermeability, was investigated. Electrochemical impedance measurements with HMECs on gold-film electrodes were conducted with two different commercial devices, xCELLigence^®^ from ACEA Biosciences, Inc. and Electric Cell-substrate Impedance Sensing (ECIS^®^) from Applied BioPhysics, Inc. The xCELLigence^®^ system displays the measurements as a so-called Cell Index (CI), whereas the ECIS^®^ device provides impedance values. The results were compared with those from a classic Transwell^®^ chamber assay using fluorescence-labeled dextran (40 kDa) as permeability tracer.

## Results

### Histamine-induced endothelial hyperpermeability was determined more reliably by impedance measurements

The autacoid histamine is mainly produced by mast cells and basophils and represents an important pro-inflammatory and pro-edematous mediator. It causes an immediate and transient activation of the actin cytoskeleton resulting in the retraction of adjacent ECs^15^. This is mediated by the activation of myosin light chain (MLC) 2, a crucial regulator of the interaction of actin and myosin (Suppl. Fig. 1A), and by the formation of F-actin stress fibers (Suppl. Fig. 1B).

**Figure 1:**
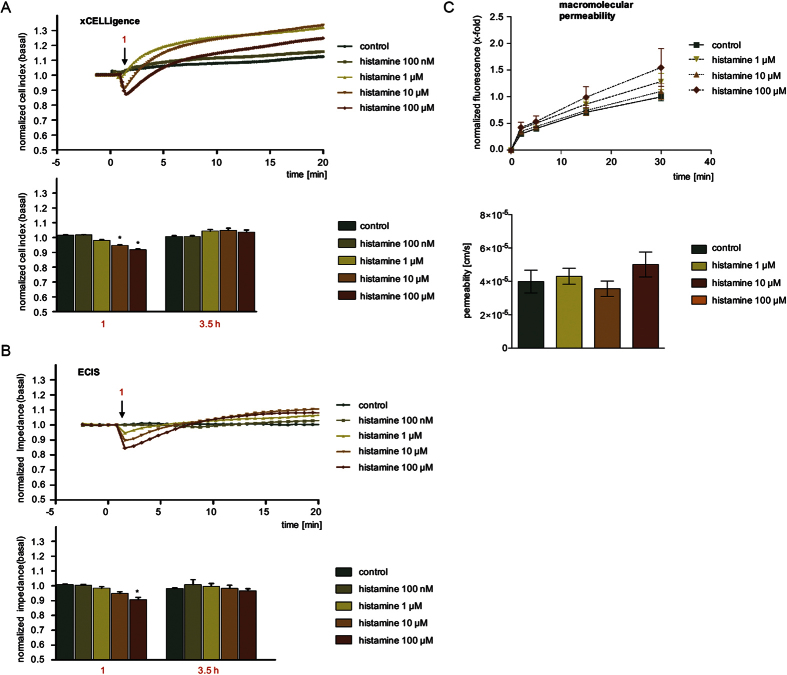
Histamine-induced endothelial hyperpermeability was determined more reliably by impedance measurements. HMECs were treated with increasing concentrations of histamine as indicated. Representative experiments show time resolved changes in normalized CI (**A**) or normalized impedance values (**B**). The bar graphs show the normalized CI (**A**) or impedance levels (**B**) of two time points for all experiments performed here (arrow 1: maximum effect of histamine). (**C**) Macromolecular permeability of FITC-dextran (40 kDa) across an HMEC cell layer was measured with a Transwell^®^ two-compartment system. Samples were taken from the lower compartment at indicated time points. The results are depicted as normalized FITC-dextran concentrations or as absolute permeability (reference time point was 30 min in each case). At least three independent experiments were performed. Data are expressed as mean ± SEM. *P ≤ 0.05 (one-way ANOVA followed by Newman-Keuls post-hoc test).

Impedance-based analysis with the xCELLigence^®^ system detected a decrease of normalized CI upon histamine addition in a concentration dependent manner within two minutes. Changes were significant for the concentrations 10 and 100 μM, whereas 100 nM did not trigger a pronounced response (Fig. 1A). Interestingly, subsequent to the initial drop, HMECs responded to histamine treatment with increasing CI values (Fig. 1A). This rebound effect occurred within the first five minutes after treatment and vanished after 3.5 h. Testing the effect of histamine with ECIS^®^ revealed the same concentration-dependent decrease of the normalized impedance (Fig. 1B), however, only 100 μM histamine evoked a response that was significantly different from untreated cells (control).

In principle, the macromolecular permeability assay (Fig. 1C, upper graph) also showed a concentration-dependent effect of histamine. However, the observed differences were small and none of the histamine concentrations could reach an effect that was statistically significant. Also the absolute permeability values (Fig. 1D, lower graph) did not significantly differ from each other.

Thus, experiments using histamine as stimulus should rely on impedance-based assays rather than on MP measurements, since the latter system is too insensitive to clearly discriminate the effects of different concentrations.

### The barrier-protecting effect of forskolin was reliably detected by all systems

Forskolin is a strong activator of the adenylyl cyclase and known to strengthen the endothelial barrier function^16^. First, we demonstrated the functionality of forskolin in HMECs. As expected, the compound was able to suppress the TRAP-induced transient phosphorylation of myosin light chain (MLC) 2 (Suppl. Fig. 2A) and the formation of F-actin stress fibers (Suppl. Fig. 2B). Forskolin alone induced a typical cortical F-actin seam, which is a sign of an intact endothelial barrier^17^.

**Figure 2:**
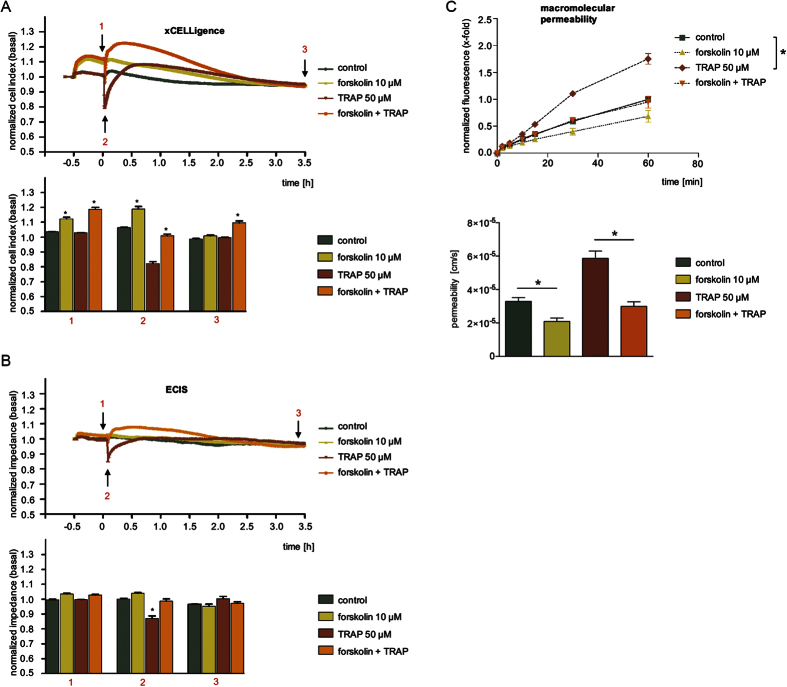
The barrier-protecting effect of forskolin was reliably detected by all systems. HMECs were treated with forskolin (10 μM) for 30 min (time point −0.5 h) and subsequently exposed to TRAP (50 μM). The time courses of compound-induced alterations of normalized CI levels (**A**) and normalized impedance values (**B**) have been normalized to control conditions. The bar graphs summarize changes in CI (**A**) or normalized impedance values (**B**) after normalization to untreated controls for all experiments at the time points indicated in A and B by black arrows (1: TRAP addition; 2: maximum effect of TRAP; 3: after 3.5 h). (**C**) Macromolecular permeability of FITC-dextran (40 kDa) across an HMEC cell layer was measured with a Transwell^®^ two-compartment system. Samples were taken from the lower compartment at indicated time points. All experiments were performed independently at least three times. The results are depicted as normalized FITC-dextran concentrations or as absolute permeability (reference time point was 60 min in each case). Data are expressed as mean ± SEM. *P ≤ 0.05 (one-way ANOVA followed by Newman-Keuls post-hoc test).

The normalized CI (Fig. 2A) and normalized impedance values (Fig. 2B) of HMECs increased immediately after the addition of forskolin at time point −0.5 h. Of note, a much stronger increase of the CI parameter (approx. 15%) was detected by the xCELLigence^®^ device (Fig. 2A) compared to the normalized impedance values (approx. 5%) measured by ECIS^®^ (Fig. 2B). After 30 min of forskolin treatment, HMECs were treated (at time point 0 h) with 50 μM TRAP in order to increase endothelial permeability^18^. Both impedance-based devices detected a rapid and significant decrease (Fig. 2A,B). Since forskolin induces an initial increase of CI and impedance values (t = −0.5 h), we normalized these curves also to the time point of TRAP addition (Suppl. Fig. 2C,D). This different normalization revealed that the maximum effect caused by TRAP was clearly attenuated by pre-treatment with forskolin in terms of its amplitude and duration (Suppl. Fig. 2E). After about 3.5 h the HMEC layers recovered and displayed CI or impedance values similar to the untreated cells (Fig. 2A,B).

In a second approach, HMECs pre-treated with forskolin (30 min) were characterized with respect to their barrier function by a MP assay. Compared to control, forskolin alone slightly decreased the passage of FITC-dextran, whereas TRAP significantly increased its accumulation in the lower compartment of the Transwell^®^ chamber at any time point of observation (Fig. 2C, upper graph). Pre-treatment of forskolin completely blocked the TRAP-induced increase of MP. The absolute permeability values at time point 60 min are given in Fig. 2C (lower graph).

Taken together, the results obtained by both impedance devices correspond well to the results of the macromolecular permeability assay for HMECs after forskolin treatment: Both types of approaches convincingly show that forskolin antagonizes TRAP-evoked cell contraction. The xCELLigence^®^ device showed more pronounced amplitudes compared to ECIS^®^ as will be discussed below.

### The ROCK inhibitor Y-27632 produced complex impedance profiles, but showed a significant barrier-protecting effect in the MP assay

Rho and its downstream effector kinase ROCK are crucial regulators of the cytoskeleton^19^ and, thus, of endothelial permeability. First, activity of the ROCK-inhibitor Y-27632 was confirmed by measuring its effects on the phosphorylation of MLC2, which was abolished despite of induction with TRAP (Suppl. Fig. 3A). Also the TRAP-evoked cytoskeletal remodeling into F-actin stress fibers was suppressed by Y-27632 (Suppl. Fig. 3B).

**Figure 3:**
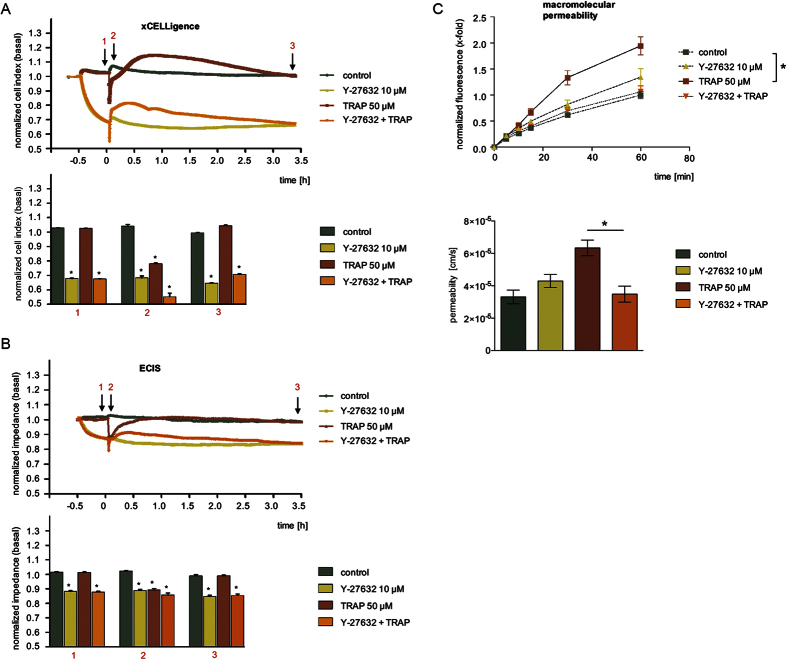
The ROCK inhibitor Y-27632 produced complex impedance profiles, but showed a clear barrier-protecting effect in the MP assay. HMECs were treated with the ROCK-inhibitor Y-27632 (10 μM) for 30 min and subsequently with TRAP (50 μM). Representative experiments show time-dependent alterations of CI (**A**) or impedance values (**B**), both normalized to untreated cells (control). The bar diagrams show the statistical analysis of all experiments for CI (**A**) or impedance changes (**B**) at time points indicated by black arrows (1: TRAP addition; 2: maximum effect of TRAP; 3: after 3.5 h) in the corresponding experiments. (**C**) Macromolecular permeability of FITC-dextran (40 kDa) across an HMEC cell layer was measured with a Transwell^®^ two-compartment system. Samples were taken from the lower compartment at indicated time points. The results are depicted as normalized FITC-dextran concentrations or as absolute permeability (reference time point was 60 min in each case). All experiments were performed independently at least three times. Data are expressed as mean ± SEM. *P ≤ 0.05 (one-way ANOVA followed by Newman-Keuls post-hoc test).

We then applied Y-27632 to HMECs and monitored the cell response with the two impedance devices: Within 30 min, the inhibitor alone strongly reduced the basal CI (Fig. 3A) and impedance values (Fig. 3B) by 30% and 15%, respectively. This initial drop was long lasting and still detectable after 3.5 h. The addition of TRAP at time point 0 h led to a further transient decrease of CI and impedance. We normalized CI values to this time point and zoomed into the time course (Suppl. Fig. 3C, left graph). Interestingly, this normalization revealed that the amplitude and duration of the TRAP effect is strongly affected by Y-27632. The inhibitor reduced the area under the curve by 85% (Suppl. Fig. 3C, right graph). All effects were less pronounced in ECIS^®^ readings compared to those recorded by the xCELLigence^®^ device.

In the MP assay, pre-treatment with Y-27632 completely prevented the TRAP-induced flux of macromolecules across endothelial monolayers (Fig. 3C, upper and lower graph). The inhibitor alone evoked a moderate, but statistically significant increase in MP. The concentration-dependency of this effect is provided in Suppl. Fig. 3D (upper and lower graph).

In summary, the ROCK inhibition strongly decreased basal values of impedance and CI. It also induced a slight increase in the MP assay. The barrier-protecting activity of the compound against TRAP-induced changes was most obvious in the MP assay. In the impedance assays the inhibitory effect of the ROCK-inhibitor was consistently masked by different starting values in presence or absence of Y-27632 at the time TRAP was added. Y-27632 could not entirely inhibit the initial and fast drop of CI/impedance induced by TRAP but values recovered faster in presence of the ROCK inhibitor. Possible reasons for the pronounced differences between the impedance versus MP readout for the inhibitor alone will be discussed below.

### The myosin inhibitor blebbistatin protected against TRAP effects in the impedance assays, but shows no effect on macromolecular permeability

The interaction of actin and myosin is essential for isometric tension and contraction of and, thus, for the regulation of endothelial barrier function. We, therefore, selected blebbistatin, an inhibitor of myosin II-ATPases, as another experimental modulator and characterized its activity. Myosin II-ATPase triggers the detachment of myosin II from actin filaments^20^. Blebbistatin reduced the TRAP-induced activation of MLC2 and F-actin stress fiber formation (Suppl. Fig. 4A).

**Figure 4:**
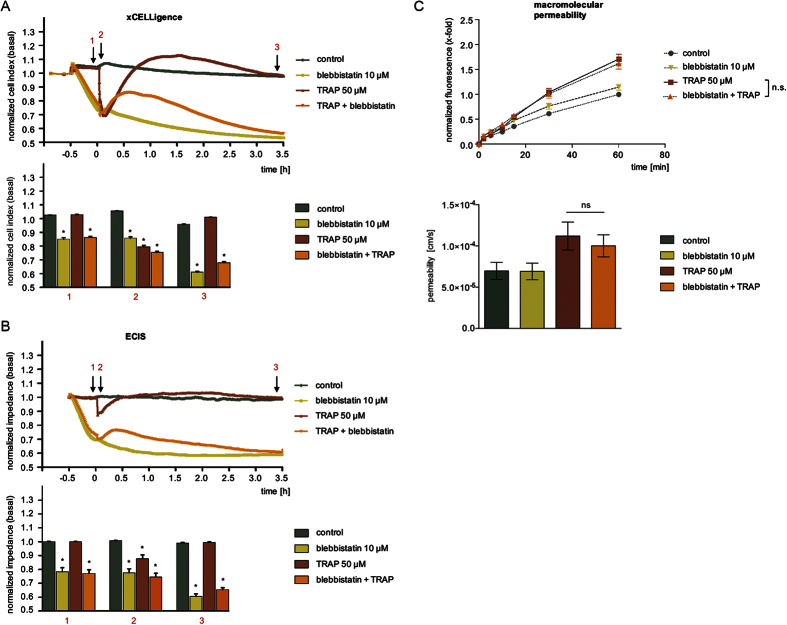
The myosin inhibitor blebbistatin protected against TRAP effects in the impedance assays, but shows no effect on macromolecular permeability. HMECs were treated with blebbistatin (10 μM) for 30 min and subsequently with TRAP (50 μM). Representative experiments indicate time-dependent changes of CI (**A**) or impedance levels (**B**), both normalized to untreated cells (control). The bar graphs summarize the statistical analysis of all experiments, normalized to control cells, for CI (**A**) or impedance measurement (**B**) at time points indicated with black arrows (1: TRAP addition; 2: maximum effect of TRAP; 3: after 3.5 h) in the corresponding experiment. (**C**) Macromolecular permeability of FITC-dextran (40 kDa) across an HMEC cell layer was measured with a Transwell^®^ two-compartment system. Samples were taken from the lower compartment at indicated time points. The results are depicted as normalized FITC-dextran concentrations or as absolute permeability (reference time point was 60 min in each case). All experiments were performed independently at least three times. Data are expressed as mean ± SEM. *P ≤ 0.05 (one-way ANOVA followed by Newman-Keuls post-hoc test).

Impedance measurements revealed that blebbistatin treatment at time point −0.5 h evoked a strong decrease of normalized CI or impedance values (Fig. 4A,B). Within 30 min, CI/impedance dropped by more than 20%. This decrease was ongoing and reached approx. 40% within 3.5 h. The application of TRAP at time point 0 h evoked an interesting effect: CI/impedance of blebbistatin-pre-treated HMECs did not drop, which means that the cells are protected against the action of TRAP, although at completely different levels of CI/impedance compared to control conditions. Moreover, TRAP induced its typical rebound effect even in the presence of blebbistatin that vanished after 3.5 h.

Contradictory results were obtained from the MP assay. Blebbistatin failed to block the TRAP-activated flux of macromolecules across the endothelial monolayer during the entire experiment (Fig. 4C, upper and lower graph). Interestingly, also the permeability under basal conditions was not altered by blebbistatin, even over a time period of 90 min (Suppl. Fig. 4B, upper and lower graph).

Thus, myosin inhibitor blebbistatin produces highly inconsistent results between MP and impedance assays: Although blebbistatin decreased basal CI/impedance values, it still potently prevented the TRAP-induced drop of both parameters. In contrast to these observations, blebbistatin did neither alter the passage of macromolecules across the endothelial monolayer significantly nor block the TRAP-induced permeabilization of the barrier.

## Discussion

Different approaches for studying endothelial permeability are available and have been reviewed comprehensively^21^,^22^,^23^. *In vivo* models often use colored probes that extravasate into the edematous tissue, *e.g*. Evans blue (Miles assay) after binding to serum albumin. Also magnetic resonance imaging or intravital microscopy techniques have been used successfully. Animal studies are of course very important and provide the most relevant and systemic insights, however, they are very laborious and not suitable for screening of large drug libraries or for the in-depth analysis of subcellular regulation processes. Therefore, *in vitro* assays using cultured endothelial cells are indispensable. Despite of their undisputed value we have to be aware that these systems suffer from missing the complete anatomic architecture of a vessel, since barrier-modulating structures and cell-types, such as pericytes, are absent. This commonly results in a much higher macromolecular permeability (10 to 100-fold) compared to the *in vivo* situation^24^. Four important methodological approaches exist to describe endothelial barrier function: determination of hydraulic conductivity, assessment of transendothelial electrical resistance (TER), flux analysis of macromolecular probes, and measurement of transendothelial impedance, which represents the newest approach in this list. Hydraulic conductivity is not easy to assess experimentally and TER measurements suffer from a lack of sensitivity for many leaky endothelial cell layers with only very low TER values. Whereas classical TER readings accurately describe the barrier function of tight endothelia like, for instance, brain capillary endothelial cells forming the blood-brain barrier^12^, they are hard to separate from the solution resistance for leaky cell layers and may be completely masked. Thus, nowadays, the most frequently used functional readout parameters for judging endothelial barrier function are macromolecular permeability and impedance.

Impedance-based monitoring of endothelial barrier function requires growth of the cells on gold-film electrodes deposited on the bottom of ordinary multi-well cell culture dishes. In contrast to filter supports, the growth substrate is, thus, not permeable. Non-invasive sinusoidal voltages in the kilo-Hertz regime are then applied to the cell-covered electrodes and the resulting current is measured. From this measurement, the impedance can be calculated and represented by a complex number^22^. The impedance of a confluent monolayer changes with the tightness of interendothelial junctions and, of note, with variations in cell morphology. These morphological changes may occur due to all kinds of cellular responses, *e.g*. in signaling processes, in cell metabolism, under flow conditions, or as a consequence of cell poisoning. Thus, the obtained data are not necessarily exclusively correlated with changes in endothelial permeability, which implies that results have to be interpreted carefully. In Transwell^®^ chambers, the cells are grown on a porous filter membrane between an upper and a lower medium-filled compartment. The flux of a labeled macromolecule across the endothelial monolayer is measured. This mimics the physiological function of the endothelium in the body, where it serves as a semi-permeable filter to solutes and plasma proteins^1^. Nevertheless, the finite pore size of the filter membranes as well as the homogenous weight of the tracer molecules is of course different from the *in vivo* situation. Fluorescence-labeled dextrans of different size are frequently employed as macromolecular tracers for permeability studies of leaky vascular endothelial cells. The molecular weight of these tracers varies between 4 and 150 kDa, however, 40 and 70 kDa dextrans are predominantly used^25^,^26^,^27^,^28^,^29^,^30^. An experimental limitation of the filter-based tracer flux approach is its cross-sensitivity to changes in cell-substratum contacts. The flux of ions and macromolecules is not only depended on interendothelial junctions, but also on the strength of cell adhesion to the substratum, which affects the size of the space available for tracer diffusion underneath the cells. Furthermore, the number and size of the pores in the filter membrane may also affect the outcome of the experiment when the filter supports are improperly selected^12^,^14^,^31^.

Given the fact that the two major readout parameters for endothelial barrier function, “impedance” and “macromolecular flux”, are physically different (besides the details mentioned above there is also the size of the permeating species, *i.e*. inorganic ions in impedance and macromolecular tracer in MP assays) it is indeed not surprising that inconsistent observations are frequent when barrier-modulating compounds are studied. This is particularly true as the cells are grown on different growth supports (permeable *vs*. impermeable), which might affect cell differentiation, polarization and nutrient supply. Nevertheless, to the best of our knowledge, we present the first direct and comparative evaluation of these two major readout parameters for one single cell type (microvascular endothelial cells) under exactly the same experimental conditions. This comparison was not intended to provide a deeper insight into the biophysical basics and implications of each method, since profound studies are available in this field^22^. It was our objective to compare the approaches from a user-oriented pharmacological perspective considering that research labs in academia and industry will apply the methods to gain knowledge about the permeability-modulating properties of bioactive compounds. We used four different substances that are well known to affect vascular permeability: histamine, forskolin, Y-27632, and blebbistatin. Moreover, TRAP was utilized to predictably induce endothelial hyperpermeability.

Histamine is known to induce a rapid and transient endothelial barrier disruption^32^. Maximum phosphorylation of MLC2 occurs already after 30 s and persists for 90 s before levels begin returning to their basal value^15^. These processes are sensitively reflected in the rapid drop of impedance with a very similar time course. Even though the MP assay also indicated a concentration-dependent effect of histamine, the curves were not significantly different. Despite the fact that the MP assay represents a cumulative measurement, *i.e*. the tracer molecules accumulate in the lower Transwell^®^ chamber, it was not sensitive enough to confirm the well-known histamine action. In this and all other experiments the two impedance approaches provided very similar time courses and only differed in the magnitude of the signal change (sensitivity). This difference in sensitivity is a consequence of the different normalization procedures and the different electrode geometries that are provided by the manufacturers. A recent report by Stolwijk *et al*. showed on the example of endothelial permeability recorded by ECIS that data normalization, sampling frequency, and electrode geometry are the major factors that determine the sensitivity of the readout when all cell-related parameters are kept constant as in this study^33^. Unfortunately, the impact of these factors varies for different cell types. Thus, there are no general design rules for optimized experimental settings that are valid across all mammalian cells. As endothelial permeability covers several orders of magnitude dependent on the origin of the endothelium, the sensitivity of the readout can be tailored by optimizing those three factors if needed. But besides these differences in details, we found both approaches to provide very similar and reproducible time courses of endothelial responses to the different pharmacological triggers.

Forskolin is a well-known endothelial barrier-protecting compound^16^. This effect was clearly visible in both, the impedance approaches and in the MP assay. The compound is even able to strengthen the barrier of otherwise untreated cells, which is reflected in the rise of impedance and the decline of MP. Interestingly, the TRAP-induced drop of impedance was visible both, under control conditions and after forskolin pre-treatment, although this drop emanated from different levels of impedance. Only normalization of the curves at the time point of TRAP addition revealed that forskolin reduced the amplitude and duration of the transient impedance drop. Thus, impedance curves must not be normalized arbitrarily, but should be carefully inspected in order to gain full information about the reaction of the endothelial cell monolayer to a certain stimulus.

Y–27632 is an inhibitor of ROCK1 (p160ROCK) and ROCK2^34^. It clearly prevented the TRAP-induced increase of macromolecular permeability. The inhibitor alone only slightly affected basal macromolecular permeability but caused a very strong decline of basal impedance values. The TRAP-triggered impedance drop occurred in untreated as well as in Y-27632-treated cells. Importantly, as with forskolin, only normalization with respect to the time point of TRAP addition revealed an inhibition of the amplitude and duration of the TRAP effect. The action of Y-27632 has been controversially discussed in the last years: One study showed that Y-27632 decreases TER values, but does not affect MP^35^. In another report, the inhibitor blocked the TRAP-induced phosphorylation of MLC2, but TER and impedance values decreased by Y-27632 treatment^36^. In accordance with our data, another study showed that Y-27632 protects against a thrombin-triggered decrease of the impedance of HUVECs^37^ and two further reports provide evidence that Y-27632 does not alter basal macromolecular endothelial permeability, even though it protects against thrombin-treatment^38^,^39^.

Blebbistatin is a specific inhibitor of the ADP release from myosin II, thus keeping it in an actin-detached conformation^40^, which results in a decreased cellular tension. In the MP assay, blebbistatin did neither affect basal permeability, nor protect against the TRAP-induced hyperpermeability. In the impedance devices, however, the compound dramatically decreased basal impedance values. It is difficult to envision for leaky endothelial cells that experimental stimulants alter the ionic but not the macromolecular permeability, this discrepancy may indicate differences in cell state that are possibly induced by the different nature of the growth substrates. Moreover, blebbistatin completely prevented a TRAP-induced drop of impedance, which might point to a protection of the barrier function. Interestingly, different actions of blebbistatin on endothelial barrier function have been communicated: The compound was found to reduce basal TER values in HUVECs^41^, but was also shown to protect against the break-down of impedance induced by the depletion of extracellular calcium in bovine corneal endothelial cells^42^.

In summary, the classic measurement of macromolecular permeability has the advantage of being easy and robust. However, as our study shows, it is insensitive to rapid and transient changes of the barrier function. Impedance measurements offer the benefit of real-time monitoring on highly different time scales (seconds to days), they are label-free, and they allow for high-throughput screening. The normalization of impedance curves and the time point of normalization are important for the interpretation of the results. Users of the commercially distributed impedance devices must be aware of these peculiarities. We conclude that both approaches can only be regarded as indicators for the complex phenomenon “endothelial permeability”. The complementary combination of the two approaches is worthwhile in order to get a better, more comprehensive and, thus, more realistic perspective on the effects of unknown compounds. Moreover, both impedance techniques have to be considered as an alternative readout in those studies addressing structure-function analysis of endothelial cell junctions when the cells are grown on impermeable supports (dishes, flasks) for biochemical or immunocytochemical experiments (western blot, immunocytochemistry). The use of permeable supports in functional assays but impermeable supports in structural studies may produce artificial conclusions.

## Methods

### Materials

Blebbistatin and histamine were purchased from Sigma-Aldrich (Taufkirchen, Germany), forskolin was purchased from Enzo Life Sciences (Lörrach, Germany), thrombin receptor-activating peptide (TRAP) was obtained from AnaSpec/MoBiTec (Göttigen, Germany), and the ROCK-inhibitor Y-27632 (hydrochloride) was purchased from Cayman Chemical/Biozol Diagnostica (Eching, Germany).

### Cell culture

Endothelial cells (ECs) were cultured in collagen G (2.5 × 10^−4^%)-coated 75 cm^2^ flasks under constant humidity at 37 °C in an atmosphere of 95% air and 5% CO_2_ in supplemented endothelial growth medium (ECGM) (PromoCell, Heidelberg, Germany) containing 10% heat inactivated fetal calf serum (FCS) (v/v), penicillin (100 U/ml)/streptomycin (100 μg/ml) (PAA, Pasching, Austria) and amphotericin B (2.5 μg/ml) (PAA). The human microvascular endothelial cell line CDC/EU.HMEC-1 (HMECs), which has been shown to retain morphologic, phenotypic, and functional characteristics of normal human microvascular endothelial cells^43^, was kindly provided by the Centers for Disease Control and Prevention (CDC, Atlanta, GA, USA) and used until the twelfth passage. Pooled primary human umbilical vein endothelial cells (HUVECs) were commercially obtained from Provitro (Berlin, Germany). HUVECs were cultivated in supplemented ECGM medium and used for experimental purposes in passage three. HMECs were used for macromolecular permeability and impedance measurement. HUVECs were exclusively used for microscopical and Western blot analysis.

### Macromolecular permeability (MP)

Transwell^®^ filter inserts (pore size 0.4 μm, 12 mm diameter, polyester membrane, Corning, New York, USA) were coated with collagen G. Subsequently, the lower compartments of the Transwell^®^ chambers were filled with 1.5 ml ECGM. HMECs suspended in 500 μl ECGM (0.125 × 10^6^ cells/well) were seeded on the upper compartment. They were grown to confluence for 24–48 h. Cells were treated as indicated with blebbistatin, forskolin, or Y-27632 for 30 min before fluorescein isothiocyanate (FITC)-dextran (40 kDa, 1 mg/ml; Sigma-Aldrich, Taufkirchen, Germany) was added to the apical side. Histamine or TRAP was added immediately after the application of FITC-dextran. 100 μl samples were taken after 0, 5, 10, 15, 30, or 60 min from the lower compartment. The removed volume was replaced by fresh medium. To avoid any inhomogenous FITC-dextran distribution in the lower compartment, Transwell^®^ plates were gently and repetitively shaken. Fluorescence (ex: 485 nm; em: 535 nm) was measured with a fluorescence plate reader (SpectraFluor Plus, Tecan, Männedorf, Switzerland). The mean fluorescence recorded from the lower compartment of untreated cells at the final time point (30 or 60 min) was set as 1. Data are expressed as relative changes compared to control levels. The absolute permeability P [cm/s] was calculated by the following equation (1)^5^,^44^:


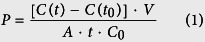


C(t) is the concentration [μg/ml] of FITC-dextran in the samples that were taken from the lower compartment after 30, 60 or 90 min (as indicated in the respective figure), C(t_0_) is the FITC-dextran concentration [μg/ml] of the samples taken after 0 min, t is the duration of the flux (s), V is the volume [cm^3^] in the lower compartment, A is the surface of the Transwell^®^ membrane [cm^2^] and C_0_ is the initial concentration [μg/ml] of the tracer on the donor side. The concentration of FITC-dextran in each sample was determined by reference to a FITC-dextran standard curve.

### Impedance measurement using xCELLigence^®^

Impedance measurement was performed with an ACEA xCELLigence^**®**^ Real-Time Cell Analyzer (RTCA) DP (distributed by Roche Diagnostics, Mannheim, Germany) consisting of an RTCA DP Analyzer, a personal computer-based RTCA control unit and single-use “E-Plates 16”. All experiments were performed following the manufacturer’s instructions. In brief, the gold-film electrodes deposited on the bottom of the “E-plate 16” electrode arrays were coated with collagen G for 30 min. Collagen solution was aspirated and replaced by 100 μl ECGM for impedance background measurements. HMECs were added at a density of 40,000 cells/well. After allowing cell sedimentation for 30 min at room temperature, E-Plates were locked into the RTCA DP Analyzer for continuous recording of impedance changes at three different AC frequencies (10 kHz, 25 kHz, 50 kHz), which are expressed as Cell Index (CI) values. CI is a dimensionless parameter based on relative impedance changes referenced to the values of the cell-free electrode at each frequency:


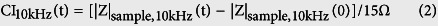



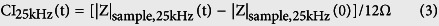



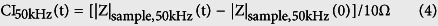


Even though the CI is measured at three frequencies, the software returns only the most sensitive reading as a function of time without further specification of how and when this selection is made. As the frequencies are rather close together, the differences between the CIs at different frequencies are supposedly small but different. Please note that CI values may go negative when the reference point |Z|_sample, freq_(t) is not equal to the impedance of the cell-free electrode. In most experiments we report on a normalized cell index. Here the cell index at any time point CI(t) of the experiment is divided by the CI value immediately before one of the test compounds is applied: Norm. CI = CI(t)/CI(t_0_). For some experiments (included in the Suppl. Figs 2,3) we have used another way of normalizing CI to highlight and emphasize certain aspects of the dataset. Here, CI(t) is normalized by subtracting the CI(t_0_) measured immediately before compound addition.

In general, CI values rise with increasing coverage of the electrode with cells, which is caused at an early stage by cell sedimentation and, later on, by cell proliferation. At constant CI levels (usually 12 to 24 h after seeding), indicating confluence of the cell monolayer, HMECs were treated with histamine alone, blebbistatin, forskolin, or Y-27632 for 30 min before TRAP was added and subsequently measured as indicated. At this stage, alterations of CI values resulted from changes in cell morphology as an indicator for changes in cell-cell and/or cell-substrate contacts. CI levels were recorded every 15 min to monitor cell growth prior to compound addition. Time resolution was set to 10 s directly before addition of compounds for an experiment time of about 3.5 h.

### Impedance measurement using ECIS^®^

All experiments were performed with an ECIS^**®**^-1600 R system (Applied Biophysics, Troy, NY, USA) in accordance with the manufacturer’s instructions. Coating of the gold-film electrodes with adhesive proteins, inoculation of the electrode arrays, and all other experimental steps were performed as described above for the xCELLigence^®^-device. The ECIS^**®**^-1600 R system consists of an ECIS^**®**^ instrument to measure complex impedance at variable frequencies, a 16-well array holder, which is stored inside the incubator, and a PC for instrument control and data storage. In brief, the growth surface of disposable 8-well electrode arrays (8w10e) were coated with collagen G for 30 min. Afterwards, the collagen solution was aspirated and replaced by ECGM for a quick impedance background check. Subsequently, HMECs were seeded in a density of 40,000 cells/well (0.75 cm^2^). After cell sedimentation and attachment to the electrode surface within 30 min at room temperature, the 8-well arrays were placed inside the ECIS^**®**^ device for impedance monitoring. All ECIS^**®**^ measurements were analyzed at an AC frequency of 32 kHz, which was identified as the most sensitive frequency for this cell type by frequency scans along an entire frequency range (10 Hz–100 kHz; compare supporting information). Exposure of endothelial cells to the various modulators was performed after reaching constant impedance values (usually 12 to 24 h after seeding) indicating confluence of the cell layer. Treatment of cells (with histamine alone or blebbistatin, Y-27632, or forskolin 30 min prior to TRAP addition) was performed analogously to the impedance measurement with the xCELLigence^®^ system. The impedance values of each well were recorded every 40 s over the entire time of analysis. It is common and well-established practice among users of the ECIS^®^ system to present impedance values as recorded along the time course of the experiment normalized to the impedance values immediately before addition of modulators or test compounds: Thus, Norm. |Z|(t) = |Z|_sample_(t)/|Z|_sample_(t_0_). Herein |Z|_sample_(t_0_) denotes the impedance magnitude immediately before test compounds were added to the cell population. Thus, in contrast to xCELLigence^®^ data the normalized impedance as reported by the ECIS^®^ device cannot go negative independent of the reference point as there is no subtraction involved.

### Statistical analysis

All data from at least three independent experiments are presented as mean ± standard error of the mean (SEM). Statistical significance was evaluated using GraphPad Prism (version 5.04, GraphPad Prism, San Diego, USA) and assessed by One-way-ANOVA and Newman-Keuls post-hoc test or unpaired t-test. Statistical differences were assumed at *P* ≤ 0.05.

## Additional Information

**How to cite this article**: Bischoff, I. *et al*. Pitfalls in assessing microvascular endothelial barrier function: impedance-based devices *versus* the classic macromolecular tracer assay. *Sci. Rep*. **6**, 23671; doi: 10.1038/srep23671 (2016).

## Supplementary Material

Supplementary Information
