# Supplementary material for: Pitfalls in assessing microvascular endothelial barrier function: impedance-based devices versus the classic macromolecular tracer assay
Source: Sci Rep. 2016 Mar 30;6:23671. doi: 10.1038/srep23671 (PMC4877919; doi:10.1038/srep23671)
Supplement: Supplementary Information [file srep23671-s1.pdf]

– SUPPLEMENTAL INFORMATION –

Pitfalls in assessing microvascular endothelial barrier function: impedance-based devices *versus* the classic macromolecular tracer assay

Iris Bischoff[1#], Michael C. Hornburger[2#], Bettina A. Mayer[2], Andrea Beyerle[1], Joachim Wegener[3], Robert Fürst[1]*

[#]contributed equally

[1]Institute of Pharmaceutical Biology, Biocenter, Goethe University Frankfurt/Main, Germany

[2]Pharmaceutical Biology, Department of Pharmacy, Center for Drug Research, University of Munich, Germany

[3]Institute of Analytical Chemistry, Chemo- and Biosensing, University of Regensburg, Germany

*Address correspondence to:

Robert Fürst, PhD

Institute of Pharmaceutical Biology, Biocenter, Goethe University Frankfurt/Main

Max-von-Laue-Str. 9, 60438 Frankfurt/Main, Germany

Phone: +49-69-798-29655; Fax: +49-69-798-763-29655

E-mail: fuerst@em.uni-frankfurt.de

Supplemental Materials and Methods

Western blot analysis

HUVECs were cultured in collagen G-coated 60 mm dishes until they reached confluence. After cell lysis and sample preparation, proteins were separated by SDS-PAGE and subsequently transferred onto nitrocellulose membranes (GE Healthcare, Munich, Germany) by electroblotting. The following antibodies were used according to manufacturer's instruction: mouse anti-actin (C4) (Millipore, Schwalbach, Germany), rabbit anti-phospho-myosin light chain (MLC) 2 (Thr18/Ser19) (Cell Signaling/New England Biolabs, Frankfurt am Main, Germany), rabbit anti-MLC2 (Santa Cruz Biotechnology, Heidelberg, Germany), HRP-conjugated goat anti-mouse IgG_1 (Biozol Diagnostica, Eching, Germany), HRP-conjugated goat anti-rabbit (Dianova, Hamburg, Germany), IRDye® 680LT-conjugated goat anti-mouse (LI-COR Biosciences, Lincoln, USA). Protein-antibody complexes were visualized by using the enhanced chemiluminescence ECL Plus Western Blotting Detection Reagent (GE Healthcare) and Super RX X-ray films (Fuji, Düsseldorf, Germany), which were developed with a Curix 60 developing system (AGFA-GAEVERT, Cologne, Germany). Actin bands were visualized with an Odyssey imaging system (LI-COR), which detects the infrared fluorescence of the IRDye® 680LT-labeled antibody.

Immunocytochemistry and confocal microscopy

HUVECs were cultured in collagen G-coated μ-slides (ibidi, Martinsried, Germany). After reaching confluence, cells were pretreated for 30 min with blebbistatin, forskolin, or Y-27632 before TRAP addition. Histamine was applied for 30 min. Untreated cells served as control. After treatment, HUVECs were fixed with 10 % formaldehyde (AppliChem, Darmstadt, Germany), washed, permeabilized with 0.2 % Triton X-100 (Sigma-Aldrich) in PBS, again washed, and subsequently blocked with 0.2 % bovine serum albumin in PBS(BSA/PBS). The cells were stained with an rabbit anti-phospho-MLC 2 (Thr18/Ser19) antibody(Cell Signaling/New England Biolabs, Frankfurt am Main, Germany) in 0.2 % BSA/PBS overnight at 4 °C. After washing three times with PBS, the cells were incubated with an AlexaFluor 488-labeled goat anti-rabbit antibody(Invitrogen, Karlsruhe,

Germany) and rhodamine-phalloidin (Invitrogen) for F-actin staining in 0.2% BSA/PBS. Images were acquired with a Zeiss LSM 510 META confocal microscope (Zeiss, Oberkochen, Germany).

Impedance analysis of cell-covered gold-film electrodes using ECIS® or xCELLigence®

The electrical tightness of cell monolayers is commonly quantified by the area-specific transendothelial or transepithelial electrical resistance (TER), given in $\Omega \cdot cm^2$. In this study we used impedance measurements of cell-covered gold-film electrodes to measure the electrical tightness of the cell layers under study, given as normalized impedance |Z| (ECIS®) or as normalized cell index CI (xCELLigence®). The numerical values of both quantities are dependent on the AC frequency that is used for data recording. Moreover, they contain contributions from the electrode/electrolyte interface and the resistance of the bulk electrolyte. Thus, the measured impedance |Z| is never identical to the TER, but it may be correlated when the measurement parameters are properly selected. In this study we have chosen 32 kHz as monitoring frequency in ECIS assays as it provides a close-to-linear correlation between the measured impedance and TER values as shown below. Since it is difficult to show the correlation between TER and |Z| experimentally, we have performed model calculations to verify the correctness of the sampling frequency:

There are two non-redundant physical models describing the impedance of cell-covered electrodes discussed in the literature. Both models describe the impedance of the electrode/electrolyte interface by a constant phase element (CPE) in series to a resistor (R_{bulk}) accounting for the solution resistance. In the simplified model (A) the cell layer is represented by an RC-element representing transendothelial resistance (TER) and capacitance.[1] The second, more precise model of the cell layer (B) comprises a non-ideal impedance element to account for the resistance arising in the cell-electrode junction. This resistance is often referred to as cleft resistance and quantified by a parameter α or α^2. The resistance of the cell-cell junctions is referred to as R_b (resistance between cells). The cell membrane is accounted for by the membrane capacitance.[2,3] The TER value from model A integrates over the contributions from R_b and α in model B.

From the pool of our experimental data we have precise knowledge about the parameter values of either model for cell layers like the ones studied here.[2,3] To verify that impedance readings at 32 kHz

are suitable for the monitoring of endothelial barrier function, we have calculated the impedance |Z|(32 kHz) as a function of TER and R_b. The capacitance of the apical and basal membrane was set to 1 µF/cm^2 as it has been found throughout the literature and from the pool of our data. This value is a direct consequence of the unfolded membrane topography in endothelial cells which will not change along the experiments performed here. The cleft resistance (model B) was set to α^2 = 25 Ωcm^2 as derived from experimental spectra.

The figure below shows the correlation between |Z|(32 kHz) and TER (Model A) as well as the correlation between |Z|(32 kHz) and the resistance between cells R_b (Model B). The data covers TERs and R_bs from 0 to 10 Ωcm^2, which is significantly more than the range of values that are relevant for our studies.

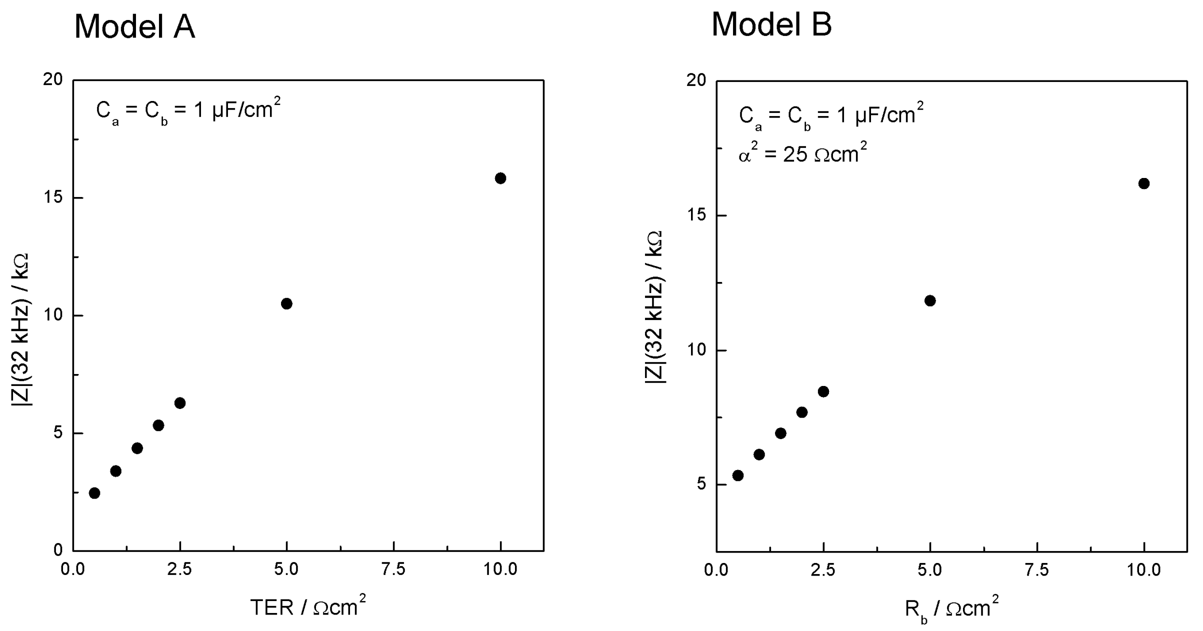

Both diagrams prove a close-to-linear correlation between TER/R_b and the impedance measured at an AC sampling frequency of 32 kHz. Numerical values of |Z| and TER are, however, not identical. The cell index CI, reported by the xCELLigence® device, reads the impedance of cell-covered electrodes at 10 kHz, 25 kHz or 50 kHz. In either case, the frequency is close enough to 32 kHz that the same conclusion applies to xCELLigence® experiments. It is noteworthy that the frequency has to be evaluated on a log scale such that 10 kHz and 50 kHz are still appropriate. The figure below shows the calculated, frequency-dependent impedance magnitude for a cell-covered ECIS® electrode as they were used in this study (blue) relative to the cell-free electrode (black). The parameters used for the

calculation are based on published values.[2,3] The impedance spectrum for cells that only spread on the electrode but do not express cell-cell junctions is shown in red for comparison. The impedance data confirms that meaningful measurements are only possible in the frequency range above 10 kHz.

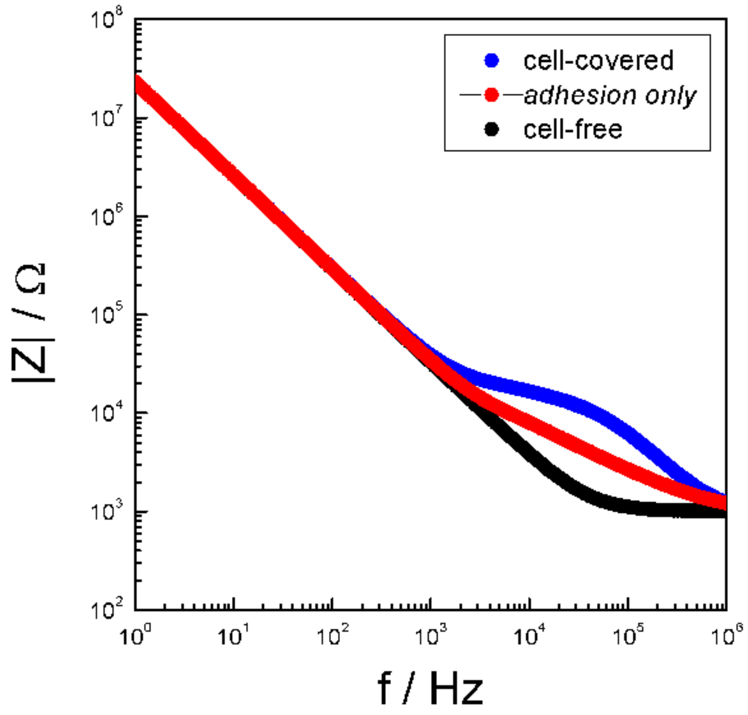

References

1 Wegener, J., Zink, S., Rosen, P. & Galla, H. Use of electrochemical impedance measurements to monitor beta-adrenergic stimulation of bovine aortic endothelial cells. *Pflugers Arch* **437**, 925-934 (1999).

2 Reiss, B. & Wegener, J. Impedance analysis of different cell monolayers grown on gold-film electrodes. *Conf Proc IEEE Eng Med Biol Soc* **2015**, 7079-7082 (2015).

3 Giaever, I. & Keese, C. R. Micromotion of mammalian cells measured electrically. *Proc Natl Acad Sci U S A* **88**, 7896-7900 (1991).

Supplemental Figure 1

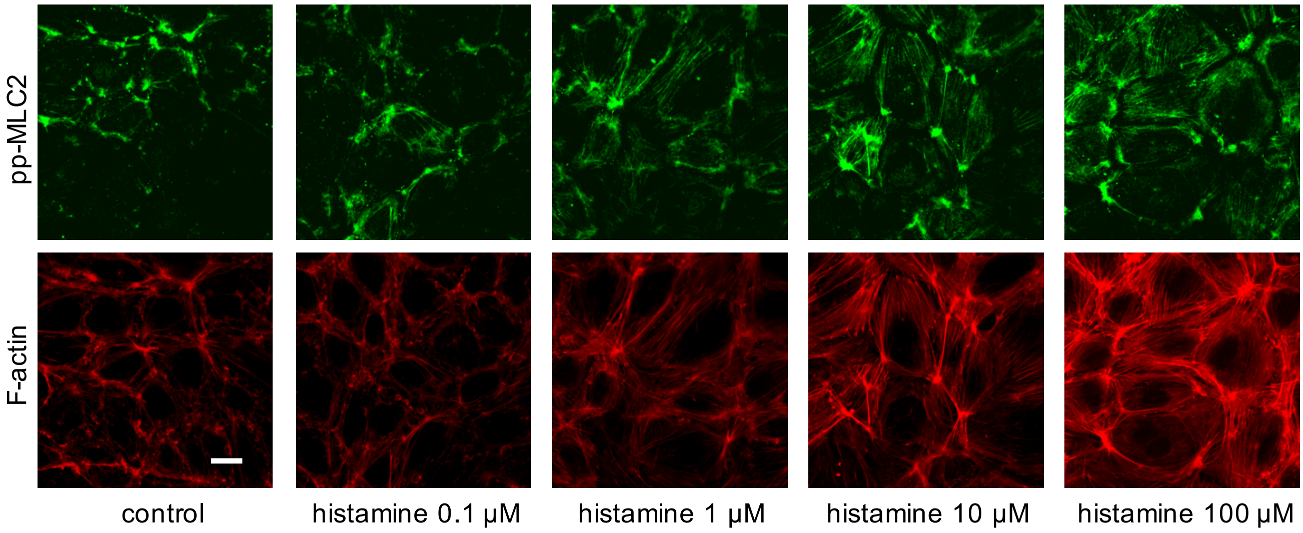

Supplemental Figure 2

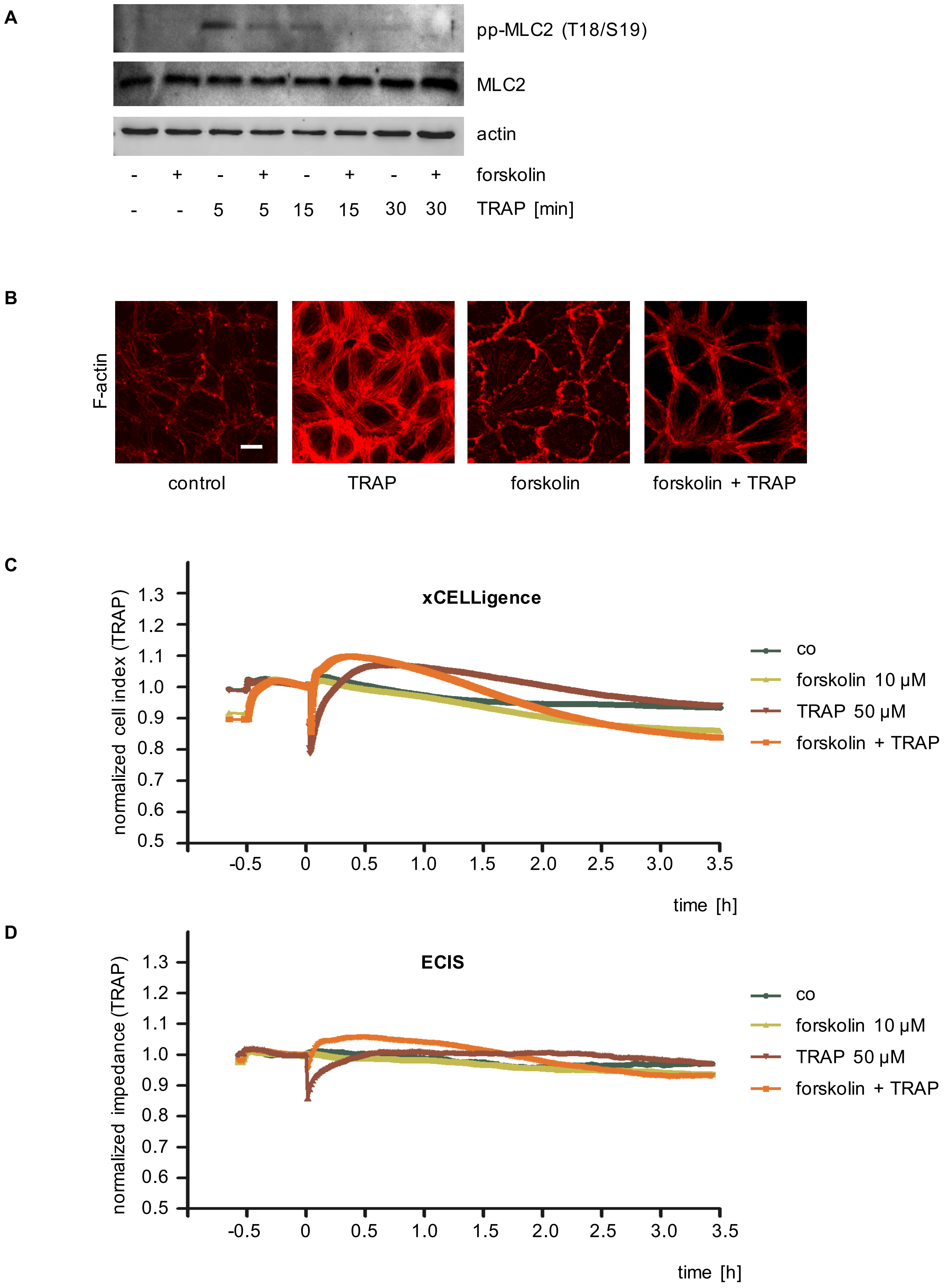

Supplemental Figure 2

E

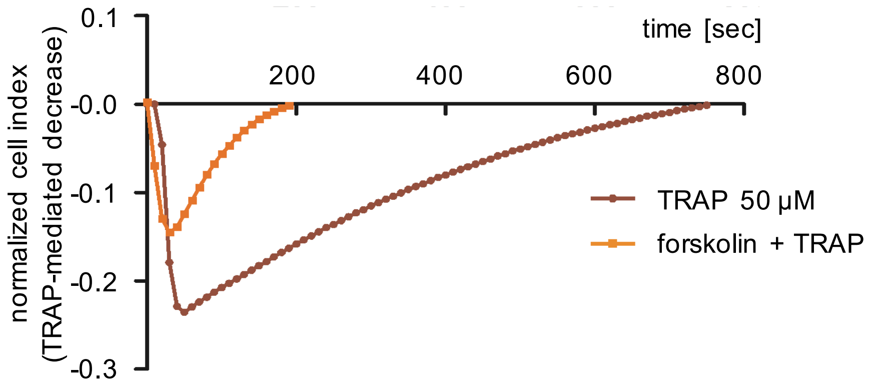

Supplemental Figure 3

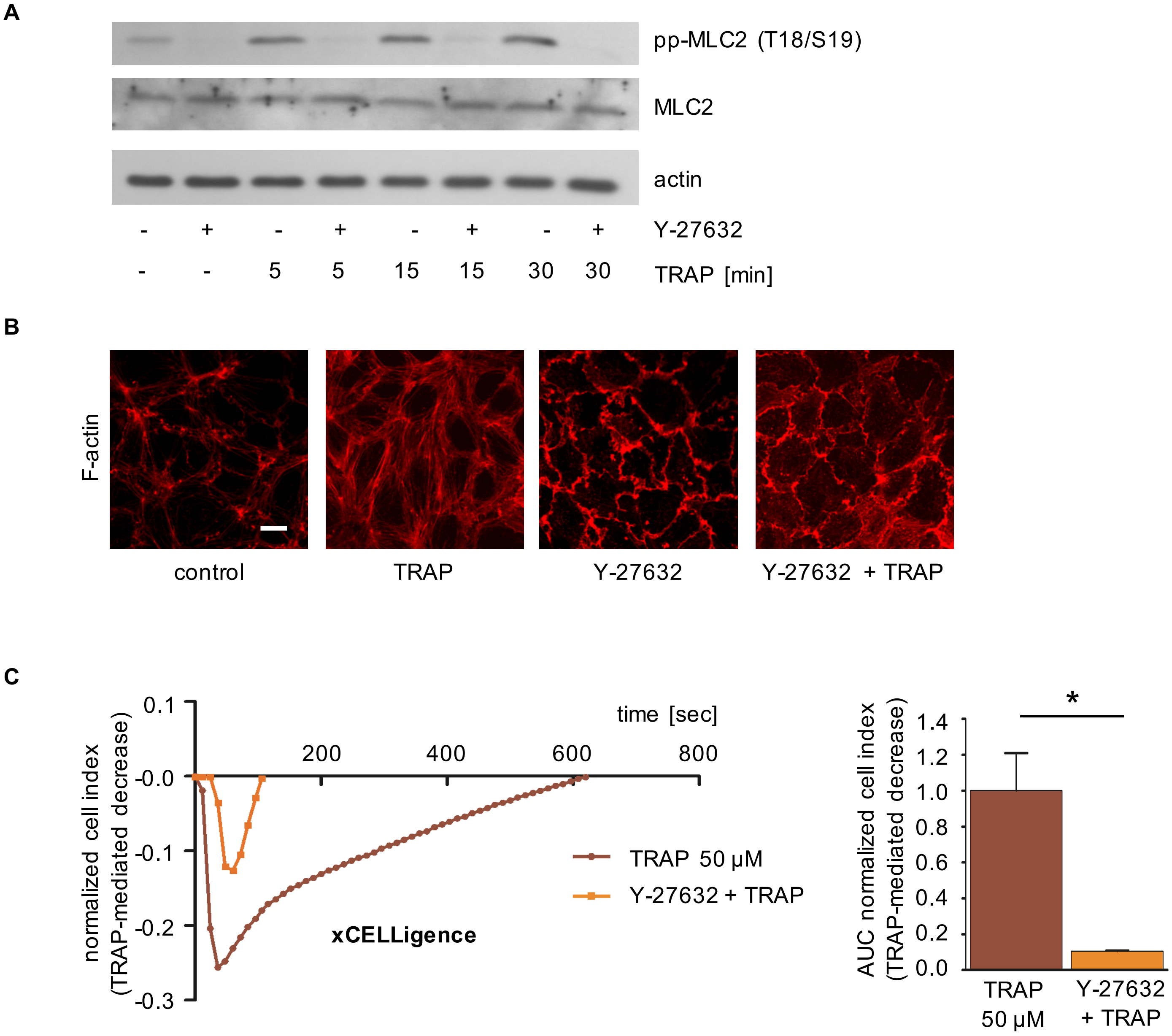

Supplemental Figure 3

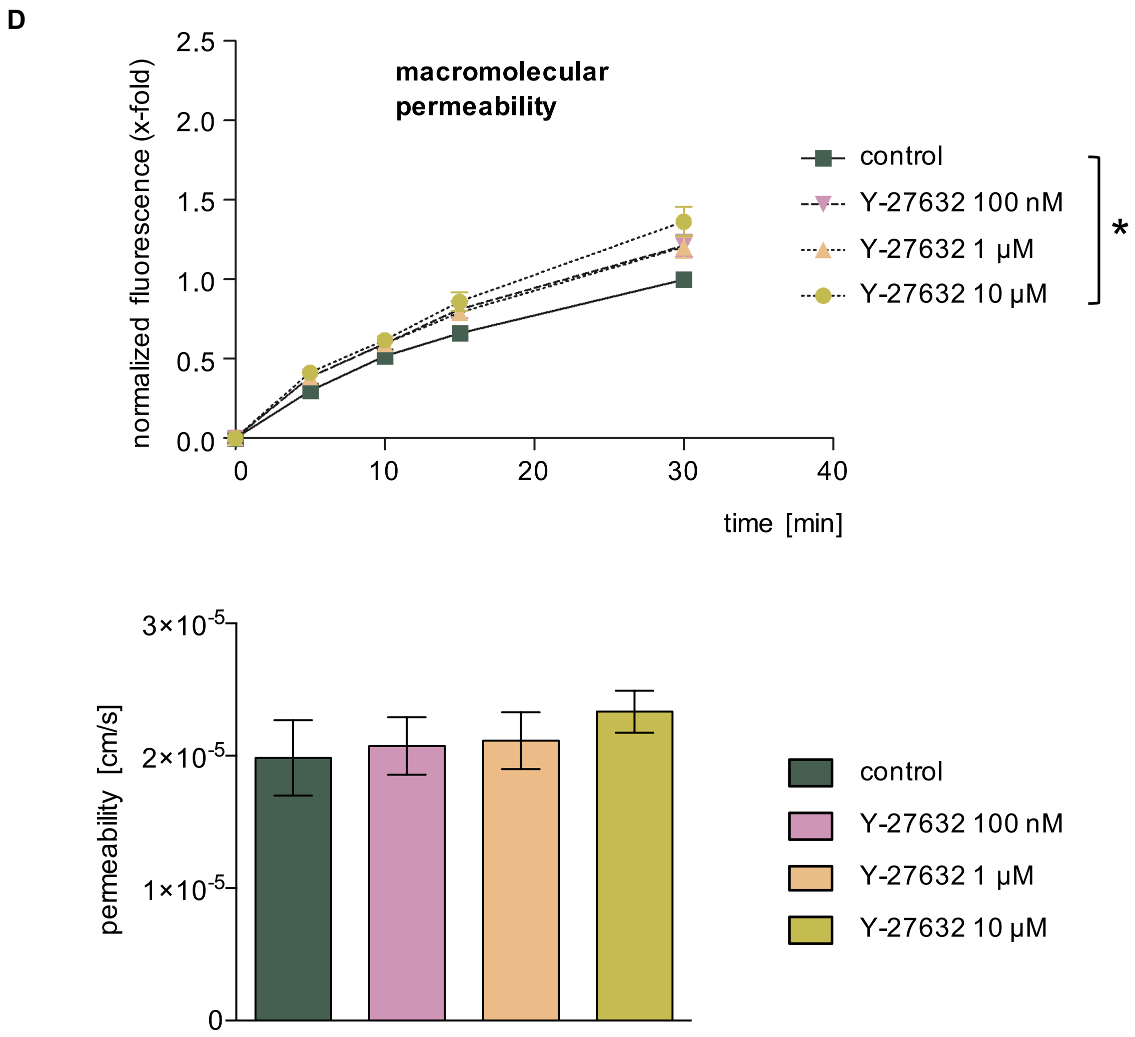

Supplemental Figure 4

A

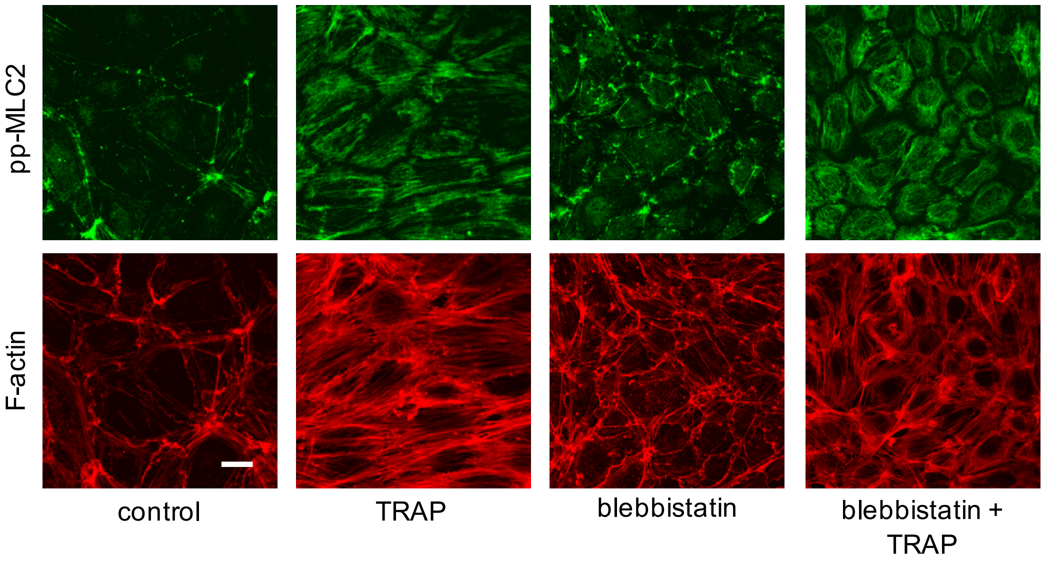

B

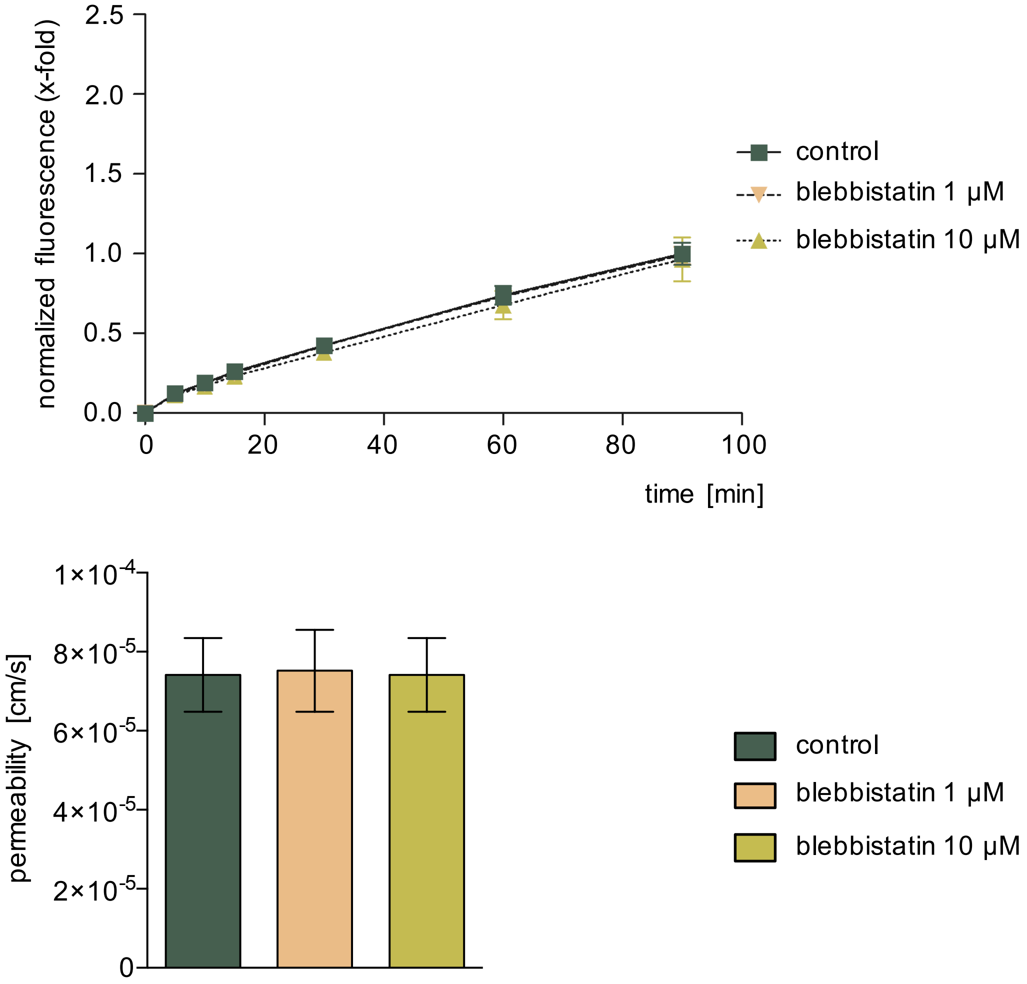

Supplemental Figure Legends

Supplemental Figure 1

Histamine. HUVECs were treated with histamine (0.1, 1, 10, 100 µM) for 30 min or remained untreated (control). Phosphorylation of the myosin light chain (MLC) 2 (Thr18/Ser19) and F-actin distribution were analyzed by immunocytochemistry and confocal microscopy. One representative set of images is shown out of three independently performed experiments. White bar = 20 µm.

Supplemental Figure 2

Forskolin. HMECs or HUVECs were treated with forskolin (10 µM) for 30 min and subsequently with TRAP (50 µM). (A) Phosphorylation of the myosin light chain (MLC) 2 (Thr18/Ser19), pan-MLC2, and β-actin was determined by Western blot analysis. (B) F-actin distribution was analyzed by immunocytochemistry and confocal microscopy. The time course of compound-induced alterations of normalized CI levels (C) and normalized impedance values (D) are displayed by representative experiments. CI and impedance values were normalized at the time point of TRAP addition (0 h). (E) A close-up of the TRAP-induced drop of the CI below control levels is shown by a representative graph. All experiments were performed independently at least three times.

Supplemental Figure 3

Y-27632. HMECs were treated with the ROCK-inhibitor Y-27632 (10 µM) for 30 min and subsequently with TRAP (50 µM). (A) Phosphorylation of the myosin light chain (MLC) 2 (Thr18/Ser19), pan-MLC2, and β-actin was determined by Western blot analysis. (B) F-actin distribution was analyzed by immunocytochemistry and confocal microscopy. (C, left) A close-up of the TRAP-induced drop of the CI below control levels is shown by a representative graph. (C, right) The bar graph shows the quantification of the respective area under the curve of all performed experiments. *$P \leq 0.05$ (unpaired *t*-test). (D) Cells were treated with the indicated concentration of Y-27632. Macromolecular permeability of FITC-dextran (40 kDa) across an HMEC cell layer was measured with a Transwell® two-compartment system. Samples were taken from the lower

compartment at the indicated time points. The results are depicted as normalized FITC-dextran concentrations or as absolute permeability (reference time point was 30 min in each case). *$P \leq 0.05$ (one-way ANOVA followed by Newman-Keuls post-hoc test). All experiments were performed independently at least three times. Data are expressed as mean ± SEM.

Supplemental Figure 4

Blebbistatin. HMECs were treated with Blebbistatin (1 or 10 µM) for 30 min and subsequently with TRAP (50 µM). (A) Phosphorylation of the myosin light chain (MLC) 2 (Thr18/Ser19) and F-actin distribution were analyzed by immunocytochemistry and confocal microscopy. One representative set of images is shown out of three independently performed experiments. White bar = 20 µm. (B) Cells were treated with the indicated concentration of blebbistatin. Macromolecular permeability of FITC-dextran (40 kDa) across an HMEC cell layer was measured with a Transwell® two-compartment system. Samples were taken from the lower compartment at the indicated time points. The results are depicted as normalized FITC-dextran concentrations or as absolute permeability (reference time point was 90 min in each case). All experiments were performed independently at least three times. Data are expressed as mean ± SEM.
